# Detection of bronchoesophageal fistula in an infant: a case report

**DOI:** 10.11604/pamj.2025.52.143.42787

**Published:** 2025-12-05

**Authors:** Aika Shoo, Frederick Lyimo, Charles Komba, Rachel Kajuna, Mary deAlmeida, Namala Mkopi, Evance Godfrey

**Affiliations:** 1 Department of Paediatrics and Child Health, Muhimbili National Hospital, Dar es Salaam, Tanzania,; 2 Department of Radiology and Diagnostic Imaging, Muhimbili National Hospital, Dar es Salaam, Tanzania,; 3 Department of Surgery, Muhimbili National Hospital, Dar es Salaam, Tanzania,; 4 Children´s Healthcare of Atlanta, Emory University, Georgia, Atlanta, United States

**Keywords:** Bronchoesophageal fistula, congenital, recurrent respiratory symptoms, case report

## Abstract

Bronchoesophageal fistula (BEF) is a rare malformation, presenting with abnormal communication between the bronchus and the esophagus, which can be either congenital or acquired. Congenital bronchoesophageal fistula is rare; this case study presents a clinical case of a six-month-old male infant of African descent with a history of chronic cough and breathing difficulties since birth, treated as a case of recurrent pneumonia without improvement. Following a thorough series of investigations, the diagnosis of bronchoesophageal fistula (BEF) was confirmed, prompting the decision to proceed with surgical intervention. This case highlights the importance of maintaining a high index of suspicion to accurately diagnose BEF.

## Introduction

Bronchoesophageal fistula (BEF) is a pathological fistulous connection between the bronchus and the esophagus. This condition can either be congenital or acquired. Acquired broncho-esophageal fistula, commonly seen in adults, develops due to lung infections such as pulmonary tuberculosis, malignancies around the esophagus and surrounding structures, trauma due to prolonged endotracheal intubation or endoscopic procedures, ingested foreign bodies, or even blunt chest injuries. Congenital forms, although they are rare compared to acquired forms, are also less frequently seen than tracheoesophageal fistulas (TEF) by a ratio of 25 to 50%. Onsets of symptoms can occur at any time from the neonatal period to adulthood [1,2]. Patients with BEF present with chronic coughing, particularly during meals, recurrent respiratory infections, and failure to thrive seen in children [3]. There is no known racial or gender predisposition associated with this condition [1]. This case report presents a case of a six-month-old infant who has experienced chronic coughing and difficulty in breathing since birth. He was initially diagnosed with recurrent pneumonia, but did not show any improvement with treatment.

## Patient and observation

**Patient information:** a six-month-old African infant delivered at term following an uneventful triplet pregnancy and with a birth weight of 3.1kg, presented with a history of chronic cough, started on the fourth day of life, seen mostly during or after breastfeeding. Several weeks after the onset of coughing, he developed difficulty in breathing and wheezing. At one month of age, recurrent episodes of fever spikes, ranging from low to high grade, also developed.

**Timeline of the current episode:** he was initially treated in multiple healthcare facilities for severe pneumonia but showed no clinical improvement. At four months of age, tracheoesophageal fistula was suspected, leading to the insertion of a nasogastric tube. However, the coughing and breathing difficulties persisted; ultimately, he was referred to our hospital at six months of age for further evaluation. No family history of atopy, open TB contact, or prolonged fever.

**Clinical findings:** upon admission, he was lethargic, malnourished with 3.5kg, and had severe respiratory distress. Physical examination revealed tachypnea (respiratory rate of 78 breaths per minute), oxygen saturation below 85% in room air, intercostal recessions, bronchial breath sounds with bilateral crepitations (more pronounced on the left lung), and normal heart sounds. Other systemic examinations were unremarkable.

**Figure 1:**
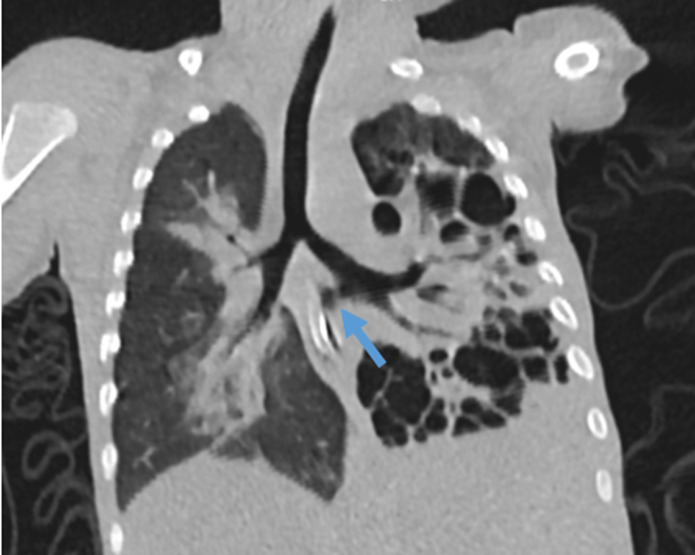
chest computed tomography coronal reformatted view showing a fistulous connection between a distal left main bronchus and the esophagus (arrow) and cystic changes

**Figure 2:**
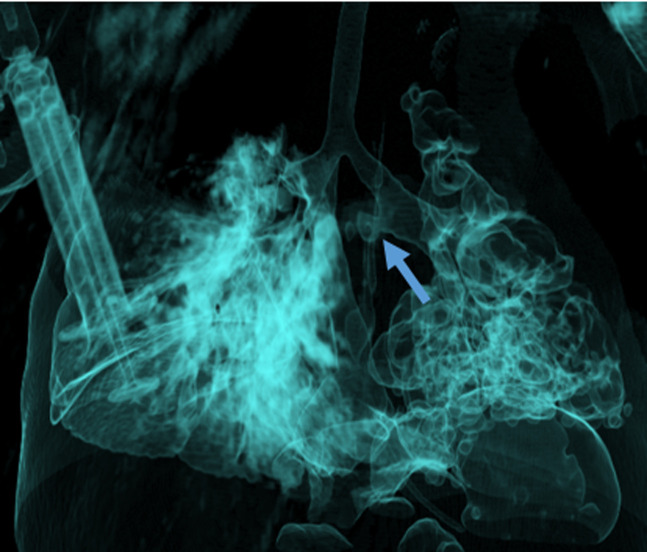
a volume-rendered computed tomography image showing fistulous connections between the distal left main bronchus and the esophagus

**Diagnostic assessment:** complete blood count results showed leukocytosis with neutrophil predominance and moderate microcytic hypochromic anemia. C-reactive protein (CRP) levels were significantly elevated. Gastric aspirate for acid-fast bacilli was negative. Chest X-ray revealed diffuse cystic changes in the left lung and consolidation in the right lung, raising concerns of a congenital lung malformation. A chest computed tomography (CT) scan was subsequently performed, confirming communication between the distal left main bronchus and the distal esophagus (Figure 1). The CT scan's volume-rendered reformatting displayed extensive cystic changes, consolidation, and a fistulous tract (Figure 2).

**Diagnosis:** based on the diagnostic findings, a diagnosis of bronchoesophageal fistula involving the distal left main bronchus and the distal esophagus, along with severe cystic changes in the left lung and a superimposed infectious process in the right lung, was established. Flexible bronchoscope would have been used, but it is not available in our setting for infants.

**Therapeutic intervention:** he was reviewed by cardiothoracic surgeons and planned for surgical resection. Intraoperatively, sequestered left lung segments containing pus were seen along with a bronchoesophageal fistula originating from the lower third of the esophagus communicating with the left main bronchus. A left pneumonectomy and fistula ligation were performed.

**Follow-up and outcome of the intervention:** he was discharged from the hospital, currently on regular clinic follow-up and his symptoms have subsided with marked improvement in his nutritional status.

**Patient´s perspective:** parents were grateful that the underlying diagnosis was found, and the procedure was done successfully.

**Informed consent:** written informed consent for publication of this case was obtained from the parents.

## Discussion

Bronchial esophageal fistula, as the name suggests, is an abnormal fistulous communication between the bronchus and the esophagus. Bronchoesophageal fistula are reported frequently in adults compared to children, commonly associated with malignancy of the chest or trauma. Diagnosing BEF can be challenging, especially in congenital forms; hence, it is often delayed or recurrently misdiagnosed due to nonspecific symptoms, including chronic cough and recurrent respiratory tract infections, as observed in many cases, including in our child. Initially, he was treated for pneumonia, but his symptoms did not improve. Subsequently, the possibility of tracheoesophageal fistula was considered, leading to the insertion of a nasogastric tube, yet the symptoms persisted, which further led to serial investigations, including a chest CT scan, which revealed a fistula connecting the distal left main bronchus and the distal esophagus [1, 4-7]. In 1967, Braimbridge and Keith classified bronchoesophageal fistulas into four types. Type I involves congenital bronchoesophageal fistulas associated with congenital esophageal diverticulum, type II is a simple bronchoesophageal fistula, type III involves bronchoesophageal fistulas with intralobar cysts, and type IV describes bronchoesophageal fistula with pulmonary sequestration. Our child was classified as BEF type IV, as evident in the CT scan [2,6] (Figure 1).

**Treatment:** the mainstay of treatment is surgical intervention, which consists of fistula excision and closure of the opening, and several reviews have shown a high rate of success, even for the case of our child. After the surgical intervention, symptoms subsided, and they are thriving well on regular outpatient clinic follow-up [7].

## Conclusion

Bronchoesophageal fistula is rare and often associated with nonspecific symptoms that may delay diagnosis; hence, a high index of suspicion is required for such rare cases, and more importantly, radiological investigations play a crucial role in accurately diagnosing such cases.

## Available online at

https://www.panafrican-med-journal.com//content/article/52/143/full
